# Adversarial robustness of deep learning-based thyroid nodule segmentation in ultrasound

**DOI:** 10.1371/journal.pdig.0001797

**Published:** 2026-11-04

**Authors:** Nicholas Dietrich, David McShannon

**Affiliations:** 1 Temerty Faculty of Medicine, University of Toronto, Toronto, Ontario, Canada; 2 Faculty of Engineering, McMaster University, Hamilton, Ontario, Canada; National University of Singapore, SINGAPORE

## Abstract

Deep learning-based segmentation models are increasingly integrated into clinical imaging workflows, yet their robustness to adversarial perturbations remains incompletely characterized, particularly for ultrasound images. We evaluated adversarial attacks and inference-time defenses for thyroid nodule segmentation in B-mode ultrasound. Two black-box adversarial attacks were developed: (1) Structured Speckle Amplification Attack (SSAA), which injects boundary-targeted noise, and (2) Frequency-Domain Ultrasound Attack (FDUA), which applies bandpass-filtered phase perturbations in the Fourier domain. Three inference-time mitigations were evaluated on adversarial images: randomized preprocessing with test-time augmentation, deterministic input denoising, and stochastic ensemble inference with consistency-aware aggregation. Experiments were conducted on a U-Net segmentation model trained on cine-clips from a database of 192 thyroid nodules. The baseline model achieved a mean Dice similarity coefficient (DSC) of 0.76 (SD 0.20) on unperturbed images. SSAA reduced DSC by 0.29 (SD 0.20), from 0.76 to 0.47, while maintaining high visual similarity (SSIM = 0.94). FDUA resulted in a smaller DSC reduction of 0.11 (SD 0.09), from 0.76 to 0.65, with lower visual fidelity (SSIM = 0.82). Against SSAA, all three defenses significantly improved DSC after correction, with deterministic denoising showing the largest recovery (+0.10, p < 0.001), followed by randomized preprocessing (+0.09, p < 0.001), and stochastic ensemble inference (+0.08, p = 0.002). No defense achieved statistically significant improvement against FDUA, although recall consistently improved at the cost of reduced precision. The tested inference-time defenses therefore partially mitigated spatial-domain perturbations, whereas statistically significant DSC recovery was not demonstrated against frequency-domain perturbations, highlighting attack-specific challenges in adversarial robustness evaluation.

## Author summary

Artificial intelligence systems are increasingly used to assist with medical image analysis, including thyroid nodule segmentation on ultrasound. These systems are typically evaluated using standard test images, yet subtle image changes can alter model behaviour. We examined how a deep learning model for thyroid nodule segmentation responds to deliberate, visually plausible image perturbations introduced under conditions where the attacker cannot access the model’s internal workings. Reference segmentations were used to evaluate worst-case vulnerability. We developed two ultrasound-specific perturbation strategies, one targeting spatial texture patterns and another affecting frequency-based image structure, and evaluated their impact on segmentation performance. We also tested three strategies that can be applied without model retraining, including image augmentation, input denoising, and aggregation of multiple predictions. We found that spatially localized perturbations could substantially degrade segmentation performance while preserving visual similarity, whereas frequency-based perturbations were less visually faithful. The evaluated strategies partially improved performance against spatial perturbations, while statistically significant recovery was not demonstrated against frequency-based perturbations. Our findings suggest that robustness depends on both imaging characteristics and perturbation structure. Although limited to one model and dataset, this study highlights the importance of evaluating model reliability beyond standard test conditions before clinical deployment.

## Introduction

Automated segmentation of thyroid nodules from B-mode ultrasound is a clinically important task that supports diagnosis, treatment planning, and longitudinal monitoring [1,2]. In recent years, deep learning approaches have shown promising performance on such tasks, enabling accurate delineation of nodule boundaries across datasets [2–4]. As these models move closer to clinical deployment, it becomes increasingly important to understand how and when they fail, particularly in settings that may compromise reliability or patient safety. At the same time, the growing integration of AI into networked diagnostic imaging workflows introduces cybersecurity concerns beyond conventional software vulnerabilities, including adversarial input manipulation and data poisoning [5,6].

Adversarial attacks, which introduce small, often imperceptible perturbations to input images that can cause substantial failures in neural network predictions, have been widely studied in natural image classification [7–9]. However, their implications for medical image segmentation remain comparatively underexplored [7,8,10]. Ultrasound imaging presents several properties that are especially relevant to adversarial manipulation. Unlike natural photographs, ultrasound images are dominated by multiplicative speckle noise, exhibit characteristic frequency-domain structure arising from beam formation, and display high inter-frame variability that may obscure subtle perturbations [11–13]. These characteristics suggest that ultrasound-specific attack strategies may differ meaningfully from generic perturbation methods and may be more difficult to detect visually.

Prior work on adversarial attacks in medical imaging has largely focused on classification tasks and has often assumed white-box access to model parameters and gradients [7,8]. In contrast, black-box attacks, where the attacker has access only to model inputs and outputs, have received less attention, particularly for ultrasound segmentation [8,14]. Existing studies involving ultrasound have primarily examined classification tasks or relied on generic pixel-level perturbations without explicitly leveraging ultrasound-specific image properties [14,15]. Segmentation tasks introduce a broader attack surface than classification, as adversarial perturbations can degrade spatial accuracy, shift predicted boundaries, or suppress detected regions entirely [8,16,17].

Equally important to characterizing attacks is the evaluation of practical defenses. Adversarial training requires retraining models and access to representative attack distributions, while certified defenses based on randomized smoothing offer formal guarantees at the cost of reduced accuracy and increased computational burden [7,18]. In contrast, inference-time defenses that preprocess inputs or aggregate stochastic predictions can be deployed without modifying the trained model and are compatible with existing architectures [7,19]. To our knowledge, the effectiveness of such lightweight defenses against ultrasound-tailored adversarial attacks has not been evaluated.

In this study, we aimed to (1) train and evaluate a baseline deep learning model for thyroid nodule segmentation in ultrasound; (2) develop two adversarial attacks tailored to ultrasound imaging under black-box query constraints; and (3) evaluate three inference-time mitigation strategies against these attacks to assess their effectiveness.

## Materials and methods

### Dataset

This study is a retrospective analysis using the publicly available single-center Stanford AIMI Thyroid Ultrasound Cine-clip database. The database is comprised of 192 biopsy-confirmed thyroid nodules from 167 patients, with B-mode ultrasound cine-clips and radiologist-annotated (by radiologists with 3–10 years of experience in thyroid ultrasound interpretation) nodule segmentation masks collected between April 2017–May 2018 [20]. All 192 nodules in the database were included without additional exclusion criteria. Each cine-clip corresponds to distinct thyroid nodules and contains multiple sequential frames. All 17,412 frames were used without subsampling. Images were resized from 802 × 1054 pixels to 256 × 256 pixels and normalized to [0, 1]. The dataset was partitioned at the patient level (70/15/15 split) to prevent data leakage, yielding 140 nodules (12,889 frames) for training, 21 nodules (1,735 frames) for validation, and 31 nodules (2,788 frames) for testing. No frames from the same patient appeared across the training, validation, and test sets. As the database was fully de-identified and publicly accessible, institutional review board approval was not required.

### Segmentation model

The target segmentation model was a U-Net [21] with an encoder of four stages (32, 64, 128, 256 features), a 512-feature bottleneck, and a symmetric decoder with skip connections. Batch normalization and ReLU activations were used throughout, with dropout (rate 0.1) during training. Training augmentations included random horizontal flips, vertical flips, and random 90° rotations. The model was trained with AdamW (lr = 1 × 10^−4^, weight decay = 1 × 10^−5^) using a combined binary cross-entropy and Dice loss, with a cosine annealing warm restarts scheduler (T_0_ = 20, T_mult_ = 2) and a batch size of 16. All experiments were implemented in Python 3.11 using PyTorch, NumPy, SciPy, and scikit-image. Training was performed on a single NVIDIA A100 80 GB GPU and completed in 26.4 minutes. Early stopping with patience of 15 epochs halted training at epoch 20, selecting the best model from epoch 5.

### Threat model

We adopted a black-box threat model in which the attacker can query the segmentation model with arbitrary input images and observe the predicted binary mask, but has no access to model weights, gradients, or architecture details. This level of access could arise in several plausible clinical settings. For example, cloud-based diagnostic platforms that accept uploaded images and return segmentation results may expose a query interface to any user with access, including legitimate users, low-privilege accounts, or external users in publicly accessible deployments. Similarly, picture archiving and communication systems integrated with artificial intelligence modules may permit indirect query access through routine clinical workflows. We intended for this set-up to reflect realistic deployment scenarios where models are accessed through inference APIs or embedded clinical devices. The attacker is permitted a fixed query budget of 500 per clip (50 iterations with a population size of 10), applied to the middle frame of each clip. The model returns only the binary segmentation mask in response to each query. The black-box designation refers to the absence of access to model parameters, gradients, or architecture, not to the use of query outputs for iterative refinement. The use of the ground truth reference for attack optimization represents an oracle-assisted evaluation intended to characterize worst-case vulnerability; a real-world attacker without a reference segmentation would require a surrogate objective such as maximizing divergence from the model’s own initial prediction. The attacker’s objective was to minimize the Dice similarity coefficient (DSC) between the model’s prediction on the perturbed image and the ground truth segmentation mask, while maintaining high visual similarity.

### Attack methods

#### Structured speckle amplification attack.

SSAA aims to exploit the multiplicative speckle noise model inherent to ultrasound by injecting spatially structured noise concentrated near the predicted segmentation boundary (Fig 1). The attack first computes the Euclidean distance transform from the boundary of the model’s current predicted mask. This predicted mask is the standard binary output returned by the model in response to each query; using it to guide subsequent perturbations is consistent with query-based black-box optimization, in which returned model outputs are used for iterative refinement, and requires no access to internal representations, gradients, or probability maps. A Gaussian weighting function, with center offset randomly sampled from 0 to 15 pixels and spatial scale (sigma) sampled from 3 to 30 pixels, determines where perturbations are strongest. Rayleigh-distributed noise, which approximates the statistical distribution of ultrasound speckle, is generated, spatially smoothed with a Gaussian kernel (sigma = 1.5), and then zero-centered and normalized to unit variance. The perturbation is applied multiplicatively to the original image with an amplitude factor randomly sampled between 0.03 and 0.20, followed by clipping to the valid intensity range [0, 1]. All SSAA attacks operated under the query constraints defined in the threat model.

**Fig 1:**
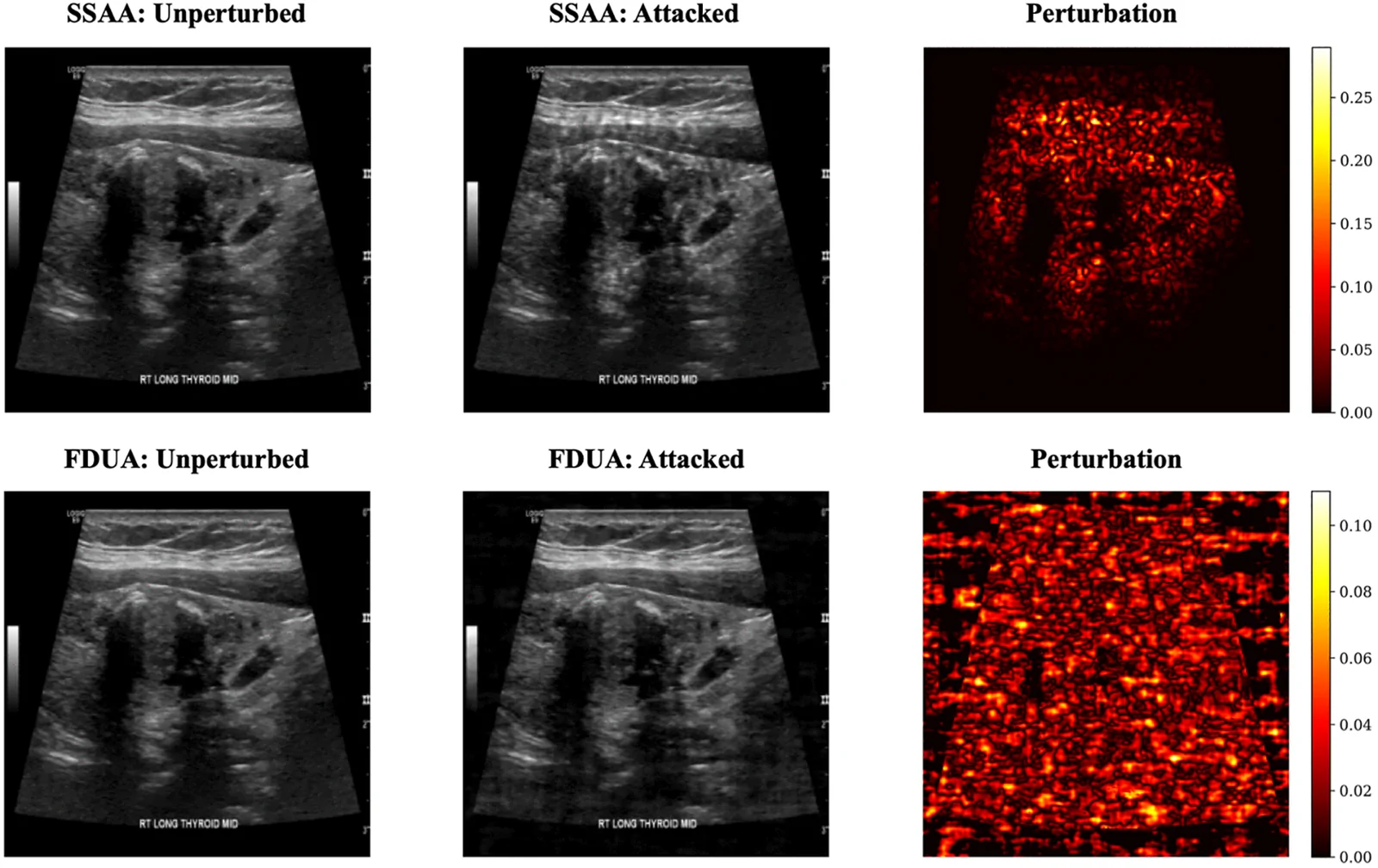
Representative examples of adversarial perturbations applied to thyroid ultrasound images. Top row = Structured Speckle Amplification Attack (SSAA). Bottom row = Frequency-Domain Ultrasound Attack (FDUA). For each attack, the unperturbed input image, adversarially perturbed image, and corresponding perturbation magnitude map are shown.

#### Frequency-domain ultrasound attack.

FDUA aimed to introduce perturbations by modifying the 2D Fourier transform of the input image within a targeted frequency band (Fig 1). The image is transformed to the frequency domain and centered. A Butterworth bandpass filter isolated a target frequency range, with the low cutoff sampled uniformly from 5 to 38 cycles per image (approximately 4% to 30% of the Nyquist frequency), the high cutoff sampled from 38 to 102 cycles per image (approximately 30% to 80% of the Nyquist frequency), and filter order randomly chosen from 1 to 4. Random phase noise uniformly distributed over [-π, π] is generated, scaled by an amplitude parameter (epsilon sampled between 0.05 and 0.50), and applied as a complex multiplicative perturbation to the bandpass-filtered frequency components. The perturbed representation is inverse-transformed to the spatial domain and clipped to valid intensity values. This approach targeted mid-frequency components where tissue texture information is concentrated. All FDUA attacks also operated under the query constraints defined in the threat model.

For both SSAA and FDUA, the attack parameter ranges were defined a priori based on visually plausible perturbation characteristics. Within these ranges, the iterative search sampled candidate parameter combinations, evaluated each against the attack objective, and retained the best-performing perturbation. The parameter ranges themselves were not optimized on the test set.

### Baseline comparison attack

To contextualize the effectiveness of the proposed ultrasound-specific attacks, we evaluated SimBA (Simple Black-box Adversarial Attacks) [22], a query-efficient black-box attack. We implemented SimBA using the pixel basis, in which candidate pixel coordinates were sampled in random order and perturbed by a fixed step size of epsilon = 0.05. The attack objective was adapted for segmentation by minimizing the DSC, consistent with the objective used for SSAA and FDUA. The positive perturbation was evaluated first; if it did not reduce DSC, the negative perturbation was evaluated, and the change was retained only when it improved the attack objective. Each coordinate evaluation required one or two model queries. SimBA was evaluated under the same query budget of 500 as the proposed attacks. Unlike SSAA and FDUA, SimBA incorporates no ultrasound-specific priors regarding speckle structure or frequency content.

### Defense strategies

Adversarial images were generated once against the undefended model and then evaluated through each defense independently. The attacker does not adapt to the presence of defenses, which reflects a realistic scenario where defenses are deployed after model training without the attacker’s knowledge. This non-adaptive approach is consistent with the methodology used in prior adversarial robustness studies in radiology [7,8], although adaptive attacks specifically designed to circumvent the tested defenses may perform differently. All defense hyperparameters were fixed a priori and were not tuned on the test data.

#### Randomized preprocessing with test-time augmentation.

This defense applies K = 5 random transformations to the input before inference and averages the resulting probability maps. Each transformation consists of a random spatial rescaling (factor sampled uniformly between 0.9 and 1.1, followed by center cropping or padding to restore the original size) and a random Gaussian blur (sigma sampled uniformly between 0.3 and 1.5). The averaged probability map is thresholded at 0.5 to produce the final binary prediction.

#### Deterministic input denoising.

This defense applies a fixed preprocessing pipeline consisting of a Gaussian blur with sigma = 1.0 followed by a 3 by 3 median filter before standard model inference. This removes high-frequency perturbation components while preserving coarser structural information needed for segmentation. As the only defense without stochastic components, it is deterministic and requires only a single forward pass. A representative frequency-domain visualization of unperturbed, FDUA-attacked, and denoised images is shown in S1 Fig.

#### Stochastic ensemble with consistency-aware aggregation.

This defense generates K = 5 augmented copies of the input, each subjected to random spatial shifts (uniformly sampled between -4 and +4 pixels with reflective boundary padding), random rescaling (factor between 0.93 and 1.07), random Gaussian blur (sigma between 0.2 and 1.2), random additive Gaussian noise (standard deviation between 0.005 and 0.02), and random brightness shift (between -0.03 and +0.03). Each copy is passed through the model to produce a probability map. A majority vote across the K predictions determines a preliminary consensus mask. Each prediction is then weighted by its pixel-wise agreement with the majority, such that pixels with unanimous agreement receive high weight and pixels with disagreement receive low weight. The final prediction is the weighted average of all probability maps, thresholded at 0.5. Representative per-pixel agreement (consistency) maps across the K = 5 augmented predictions are shown in S2 Fig.

### Evaluation metrics

Segmentation quality was assessed using the DSC, intersection-over-union (IoU), precision, recall, pixel accuracy, and the 95th percentile Hausdorff distance (HD95). Visual similarity of adversarial perturbations was quantified using the structural similarity index (SSIM), L_2_ norm, and L-infinity norm. For each mitigation, the recovery rate was computed as the fraction of the attack-induced Dice loss restored by the defense: (mitigated Dice minus undefended Dice) divided by (unperturbed Dice minus undefended Dice), and averaged across test images. The unperturbed performance cost of each defense was measured by evaluating it on unattacked images.

### Statistical analysis

All comparisons between defended and undefended conditions used a paired design, as the same 31 adversarial images (one per test nodule) were evaluated under each condition. The one-sided Wilcoxon signed-rank test was used to assess whether each defense significantly improved the adversarial DSC. The DSC was designated as the primary metric, with six comparisons (three defenses by two attacks) corrected using the Bonferroni method (adjusted α = 0.05/6 = 0.0083). SimBA was included as a secondary comparison; its three defense comparisons were corrected separately (adjusted α = 0.0167). IoU, precision, recall, and pixel accuracy were designated as secondary metrics. Secondary metric analyses were exploratory, and associated p-values were not adjusted for multiple comparisons. Effect sizes are reported as Cohen’s d computed on paired differences, with interpretation thresholds of less than 0.2 (negligible), 0.2 to 0.5 (small), 0.5 to 0.8 (medium), and 0.8 or greater (large). An illustrative post-hoc power analysis was performed for the observed FDUA defense effect sizes using n = 31 and the Bonferroni-adjusted α = 0.0083. Bootstrap 95% confidence intervals (1,000 resamples) are reported for all DSC improvement estimates.

## Results

### Baseline model performance

On the 31 test cine-clips, the U-Net model achieved a mean DSC of 0.76 (SD 0.20), IoU of 0.65 (SD 0.23), precision of 0.81 (SD 0.25), recall of 0.76 (SD 0.19), pixel accuracy of 0.95 (SD 0.04), and HD95 of 32.88 (SD 26.88) pixels. The high variability in HD95 was driven primarily by a single outlier clip with a small, fragmented nodule mask. Table 1 provides the summary of segmentation metrics across all conditions.

**Table 1:** Summary of segmentation metrics across all conditions.

Condition	DSC	IoU	Precision	Recall	Pixel Accuracy	HD95
Unperturbed (baseline)	0.76 (0.20)	0.65 (0.23)	0.81 (0.25)	0.76 (0.19)	0.95 (0.04)	32.88 (26.88)
Unperturbed + Randomized Preprocessing	0.76 (0.20)	0.65 (0.23)	0.78 (0.25)	0.79 (0.17)	0.95 (0.04)	37.05 (27.65)
Unperturbed + Denoising	0.76 (0.19)	0.64 (0.22)	0.76 (0.24)	0.81 (0.14)	0.95 (0.03)	40.84 (28.00)
Unperturbed + Stochastic Ensemble	0.76 (0.19)	0.65 (0.22)	0.78 (0.24)	0.79 (0.15)	0.95 (0.04)	37.26 (28.25)
SSAA (undefended)	0.47 (0.27)	0.35 (0.23)	0.58 (0.33)	0.52 (0.33)	0.92 (0.04)	47.64 (17.08)
SSAA + Randomized Preprocessing	0.55 (0.23)	0.42 (0.22)	0.62 (0.29)	0.62 (0.28)	0.92 (0.04)	48.31 (23.72)
SSAA + Denoising	0.57 (0.22)	0.43 (0.21)	0.60 (0.28)	0.66 (0.25)	0.92 (0.04)	49.55 (24.00)
SSAA + Stochastic Ensemble	0.55 (0.24)	0.41 (0.22)	0.62 (0.30)	0.61 (0.29)	0.92 (0.04)	49.10 (25.99)
FDUA (undefended)	0.65 (0.24)	0.53 (0.24)	0.71 (0.28)	0.68 (0.25)	0.94 (0.04)	40.89 (25.54)
FDUA + Randomized Preprocessing	0.67 (0.21)	0.54 (0.23)	0.68 (0.28)	0.74 (0.19)	0.94 (0.04)	43.74 (27.24)
FDUA + Denoising	0.67 (0.22)	0.54 (0.24)	0.67 (0.28)	0.75 (0.20)	0.94 (0.04)	48.46 (27.78)
FDUA + Stochastic Ensemble	0.66 (0.22)	0.53 (0.22)	0.69 (0.28)	0.71 (0.21)	0.94 (0.04)	43.28 (25.96)

Values reported as mean (SD). DSC = Dice similarity coefficient; IoU = Intersection over Union; HD95 = 95th percentile Hausdorff distance; SSAA = Structured Speckle Amplification Attack; FDUA = Frequency-Domain Ultrasound Attack; SD = standard deviation.

### Attack effectiveness

Fig 2 shows representative qualitative examples of segmentation outputs under unperturbed, adversarially attacked, and defended conditions for both SSAA and FDUA. SSAA reduced the mean DSC from 0.76 to 0.47 (SD 0.27), a mean DSC drop of 0.29 (95% CI [0.22, 0.36], p < 0.001, d = 1.46), while maintaining high visual similarity (SSIM = 0.94, L_2_ = 5.9, L_**∞**_ = 0.27). The attack showed substantial variability, with individual clip DSC drops ranging from 0.04 to 0.78. The distribution of adversarial DSCs across attacks and defense conditions is shown in S3 Fig. Precision decreased from 0.81 to 0.58 (SD 0.33) and recall from 0.76 to 0.52 (SD 0.33), indicating that SSAA caused both missed nodule tissue (false negatives) and spurious predictions (false positives).

**Fig 2:**
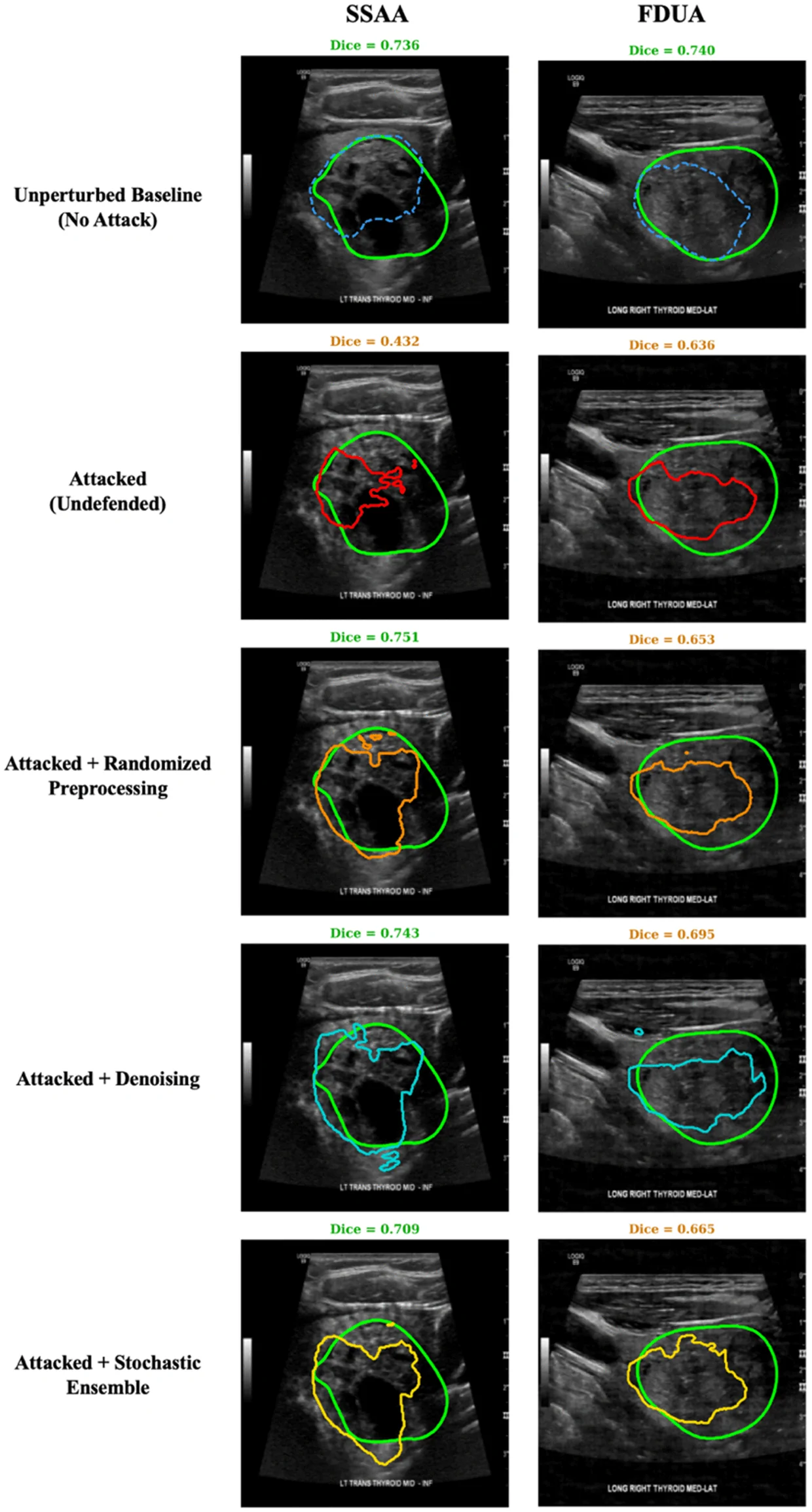
Visual examples of segmentation from unperturbed to attacked to defended for representative test cases. Left image = Structured Speckle Amplification Attack (SSAA); Right image = Frequency-Domain Ultrasound Attack (FDUA); Green contour = ground truth; Blue dashed = unperturbed prediction; Red = undefended attacked prediction; Orange = randomized preprocessing; Cyan = deterministic denoising; Yellow = stochastic ensemble.

FDUA produced a smaller mean DSC reduction from 0.76 to 0.65 (SD 0.24), a mean DSC drop of 0.11 (95% CI [0.08, 0.14], p < 0.001, d = 1.16), with lower visual fidelity (SSIM = 0.82, L_2_ = 5.0, L_**∞**_ = 0.09). Precision was relatively preserved at 0.71 (SD 0.28) while recall dropped to 0.68 (SD 0.25), suggesting that FDUA primarily caused the model to under-segment the nodule rather than produce entirely erroneous predictions. Quantitative image-similarity metrics for each attack are reported in S1 Table.

SimBA, the generic black-box baseline, produced a mean DSC reduction of 0.01 (SD 0.01), from 0.76 to 0.75 (SD 0.21), with near-perfect visual fidelity (SSIM > 0.99, L_2_ = 0.29, L_**∞**_ = 0.05). This reduction was substantially smaller than both SSAA (0.29) and FDUA (0.11). No defense achieved statistically significant improvement in DSC against SimBA (all p > 0.3). Full SimBA results are reported in S2 Table.

### Unperturbed performance cost of defenses

All three defenses imposed negligible cost on unperturbed image segmentation. Randomized preprocessing with test-time augmentation reduced unperturbed DSC by 0.0026, from 0.7601 to 0.7575. Deterministic input denoising reduced unperturbed DSC by 0.0031, from 0.7601 to 0.7570. The stochastic ensemble with consistency-aware aggregation produced a marginal improvement of 0.0005, from 0.7601 to 0.7606. Table 2 shows that all defenses imposed negligible performance cost on unperturbed images.

**Table 2:** Unperturbed segmentation performance (no attack) with each defense, showing the cost of defense on unattacked images.

Condition	Unperturbed DSC	SD	DSC Cost
Undefended (baseline)	0.7601	0.2036	—
Randomized Preprocessing	0.7575	0.2027	+0.0026
Input Denoising	0.7570	0.1850	+0.0031
Stochastic Ensemble	0.7606	0.1863	−0.0005

DSC = Dice similarity coefficient; SD = standard deviation; DSC Cost = change in Dice similarity coefficient relative to the undefended baseline (positive values indicate reduced performance).

### Randomized preprocessing with test-time augmentation

Against SSAA, randomized preprocessing significantly improved the mean DSC from 0.47 to 0.55, a gain of +0.09 (95% CI [+0.04, + 0.14], p = 0.0003, Cohen’s d = 0.61). The DSC recovery rate was 29.29%. Among secondary endpoints, IoU improved by +0.07 (p < 0.001, d = 0.62), recall improved by +0.10 (p < 0.001, d = 0.72), precision improved by +0.04 (p = 0.14, d = 0.27), and pixel accuracy improved by +0.005 (p = 0.07, d = 0.27).

Against FDUA, randomized preprocessing did not significantly improve DSC (+0.016, 95% CI [-0.03, + 0.07], p = 0.36, d = 0.11). Despite the negligible DSC improvement, this defense increased recall by +0.06 (p = 0.023, d = 0.36), but precision decreased by 0.03 (p = 0.89). This opposing recall-precision pattern explains why the net DSC effect was negligible. The DSC recovery rate was 16.92%. Table 3 reports the statistical significance and effect sizes of DSC improvements for each defense–attack pairing.

**Table 3:** Statistical significance of defense improvements on adversarial Dice similarity coefficient (DSC).

Attack	Defense	Δ DSC	95% CI	p-value	Cohen’s d	Recovery (%)
SSAA	Randomized Preprocessing	+0.09	[+0.04, +0.14]	0.0003	+0.61	29.29
SSAA	Denoising	+0.10	[+0.06, +0.15]	0.0002	+0.73	35.57
SSAA	Stochastic Ensemble	+0.08	[+0.04, +0.12]	0.0017	+0.61	28.24
FDUA	Randomized Preprocessing	+0.02	[–0.03, +0.07]	0.3603	+0.11	16.92
FDUA	Denoising	+0.01	[–0.03, +0.05]	0.1836	+0.14	20.60
FDUA	Stochastic Ensemble	+0.01	[–0.02, +0.04]	0.2647	+0.10	8.06

Significance assessed by one-sided paired Wilcoxon signed-rank test with Bonferroni correction (adjusted α = 0.0083 for six comparisons). CI = bootstrap 95% confidence interval (1,000 resamples); Recovery = percentage of attack-induced DSC loss restored by the defense.

### Deterministic input denoising

Against SSAA, deterministic input denoising achieved the largest DSC improvement among all defenses, increasing the mean DSC from 0.47 to 0.57, a gain of +0.10 (95% CI [+0.06, + 0.15], p = 0.0002, Cohen’s d = 0.73). The DSC recovery rate was 35.57%. Among secondary endpoints, IoU improved by +0.09 (p < 0.001, d = 0.76), and recall showed the largest improvement across all defense-attack combinations at +0.14 (p < 0.0001, d = 0.92). Precision improved by +0.03 (p = 0.12, d = 0.22). Pixel accuracy improved by +0.006 (p = 0.046, d = 0.31).

Against FDUA, deterministic input denoising did not significantly improve DSC (+0.015, 95% CI [-0.03, + 0.05], p = 0.18, d = 0.14). However, this defense increased recall by +0.07 (p < 0.001, d = 0.76), the strongest recall recovery among all defenses against FDUA, corresponding to a recall recovery rate of 87.65%. Precision decreased by 0.04 (p = 0.99, d = -0.40), offsetting the recall gain in the DSC. The DSC recovery rate was 20.60%.

### Stochastic ensemble with consistency-aware aggregation

Against SSAA, the stochastic ensemble improved the mean DSC from 0.47 to 0.55, a gain of +0.08 (95% CI [+0.04, + 0.12], p = 0.0017, Cohen’s d = 0.61). The DSC recovery rate was 28.24%. Among secondary endpoints, IoU improved by +0.07 (p = 0.0012, d = 0.63), recall improved by +0.09 (p < 0.001, d = 0.75), and precision improved by +0.04 (p = 0.13, d = 0.21). Pixel accuracy improved by +0.004 (p = 0.06, d = 0.19).

Against FDUA, the stochastic ensemble did not significantly improve DSC (+0.009, 95% CI [-0.02, + 0.04], p = 0.26, d = 0.10). Recall improved by +0.03 (p = 0.035, d = 0.42), while precision decreased by 0.02 (p = 0.82, d =- 0.19). The DSC recovery rate was 8.06%, the lowest among all defenses against FDUA. Fig 3 summarizes the effects of each defense across all attacks using multiple segmentation metrics, including DSC, IoU, precision, and recall.

**Fig 3:**
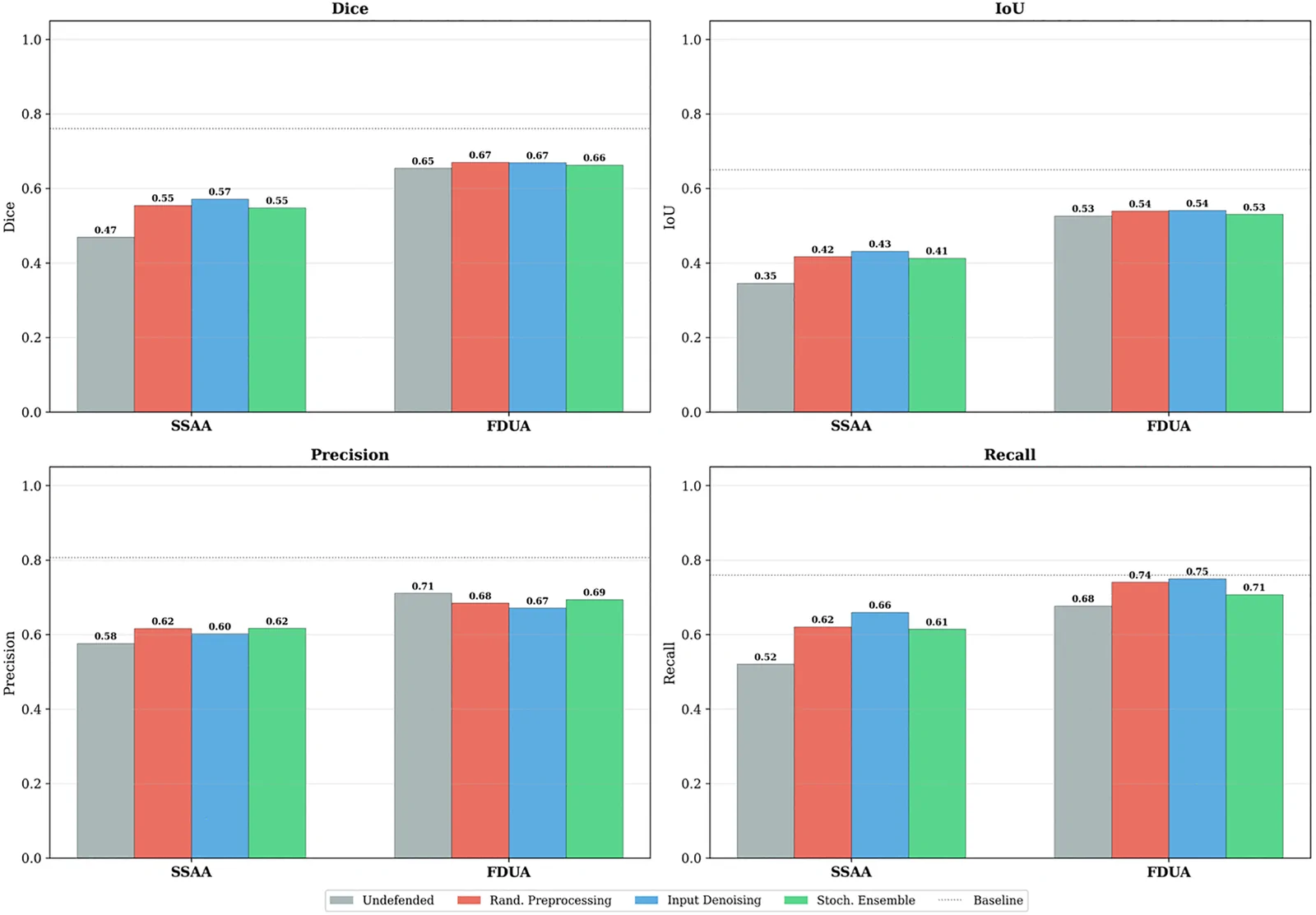
Multi-metric comparison across all attack and defense conditions. Dice = Dice similarity coefficient; IoU = Intersection over Union; SSAA = Structured Speckle Amplification Attack; FDUA = Frequency-Domain Ultrasound Attack.

### Cross-defense comparison and recovery rates

Against SSAA, all three defenses achieved comparable DSC recovery (28.24% to 35.57%), with deterministic input denoising producing the highest recovery. IoU recovery closely paralleled DSC at 26.73% to 33.48%. The defenses primarily recovered missed nodule tissue rather than correcting spurious predictions, as recall recovery rates (26.52% to 35.66%) substantially exceeded precision recovery rates (-4.63% to 8.45%). Pixel accuracy recovery ranged from 15.29% to 18.00%.

Against FDUA, DSC recovery rates were lower and not statistically significant (8.06% to 20.60%). A pattern emerged in which recall recovery rates were disproportionately higher (23.72% for the stochastic ensemble, 62.60% for randomized preprocessing, and 87.65% for deterministic denoising) while precision recovery was consistently negative (-19.97% to -29.26%). Recovery rate analyses across all metrics are provided in S3 Table and S4 Fig.

## Discussion

This study shows that a U-Net model for thyroid ultrasound segmentation can be affected by black-box adversarial perturbations that preserve visual similarity, and that the performance of the evaluated inference-time defenses varies depending on the domain in which the attack operates. In particular, SSAA showed partial mitigation with standard preprocessing, whereas no statistically significant DSC recovery was demonstrated against FDUA.

Even under a constrained query budget, the attacks were able to meaningfully degrade segmentation performance. For SSAA, the mean DSC decreased from 0.76 to 0.47, a reduction that could correspond to clinically relevant underestimation or displacement of a thyroid nodule. Importantly, these perturbations were visually subtle, making them potentially difficult to detect through routine image review. These findings demonstrate substantial model vulnerability under constrained black-box query access in an oracle-assisted stress-test setting. The increasing adoption of cloud-based artificial intelligence diagnostic platforms and networked imaging infrastructure expands the potential attack surface. Healthcare and radiology systems are already exposed to substantial cybersecurity risk because of their reliance on interconnected digital infrastructure and the sensitivity of clinical data, while the incorporation of AI introduces additional model-specific vulnerabilities such as adversarial input manipulation and data poisoning [5,6].

Moreover, a DSC of 0.47 under SSAA reflects substantial disagreement between the predicted and reference nodule contours. Depending on the spatial pattern of the error, this degree of segmentation degradation could affect derived measurements such as nodule dimensions or volume, potentially influencing longitudinal assessment or size-dependent management thresholds. The present study did not directly evaluate downstream clinical decisions. The query budget of 500 used in this study reflects an automated evaluation scenario; in practice, rate limiting and access controls would constrain query volume, though automated scripts interacting with unprotected inference endpoints could feasibly execute this volume. Accordingly, the present experiments should be interpreted as an oracle-assisted black-box stress test of model vulnerability rather than as a direct simulation of a fully operational real-world attacker.

The observed difference in defensibility between SSAA and FDUA is consistent with prior work suggesting that defense effectiveness depends on the relationship between perturbation structure and image features used by the model [23,24]. In ultrasound, where speckle and tissue texture define much of the signal content, spatially localized perturbations may be more amenable to smoothing or denoising than perturbations embedded within frequency components that overlap with normal texture patterns [11,12,25]. While this study did not directly measure model feature sensitivity, the differential response to preprocessing supports this interpretation.

From a practical standpoint, a simple deterministic denoising pipeline recovered approximately 36% of the lost DSC under SSAA with negligible cost to unperturbed performance. Randomized preprocessing and stochastic ensemble inference also produced meaningful but smaller recovery, restoring approximately 29% and 28% of lost DSC, respectively. These gains were driven primarily by improvements in recall, indicating partial recovery of missed nodule tissue rather than correction of spurious predictions.

In contrast, none of the tested defenses produced a statistically significant improvement in DSC under FDUA. One possible explanation is that FDUA introduces perturbations that are not easily separable from normal ultrasound appearance using simple input-level preprocessing. Qualitatively, defended images remained visually distinct from unperturbed images even after denoising, suggesting that the perturbations persist despite filtering. This interpretation is supported by the exploratory secondary metric patterns observed for FDUA, where recall consistently improved across defenses while precision decreased, reflecting a shift toward over-segmentation. These opposing effects largely cancelled in the DSC, resulting in minimal net improvement. Post-hoc power analysis indicated less than 5% power to detect the observed FDUA defense effect sizes (Cohen’s d = 0.10–0.14), suggesting that the non-significant findings should be interpreted as inconclusive rather than as evidence of no defense effect. The consistent improvement in recall across all defenses against FDUA, despite the lack of net DSC improvement, may nonetheless be relevant in applications where under-segmentation is considered more consequential than modest over-segmentation.

Together, these findings indicate that the evaluated input-level preprocessing strategies did not provide robust DSC recovery against FDUA under the tested conditions. The differential response observed between FDUA and SSAA highlights the importance of evaluating adversarial defenses across distinct perturbation structures rather than assuming that a single defense will generalize across attack types [7,10]. The comparison with SimBA provides additional context for the proposed attacks. At the same query budget, SimBA reduced DSC by only 0.01, compared to 0.29 for SSAA and 0.11 for FDUA, suggesting that generic pixel-level perturbations may be substantially less effective than modality-specific strategies under the evaluated conditions.

The generalizability of both the attacks and defenses to other imaging environments warrants consideration. Ultrasound image appearance is strongly influenced by vendor-specific beamforming algorithms, probe frequency, and proprietary post-processing pipelines, and domain shift across scanners and institutions is a well-recognized challenge for deep learning in ultrasound [13]. The Stanford AIMI dataset was acquired at a single institution, and the speckle patterns, frequency content, and tissue contrast in these images may differ from those acquired on other platforms. As SSAA exploits the statistical properties of speckle and FDUA targets mid-frequency texture structure, both attacks are inherently sensitive to these imaging characteristics, and their effectiveness on data acquired under different conditions remains to be established.

Several directions for future work follow from this study. The limited effectiveness of preprocessing against FDUA motivates investigation of training-time strategies that expose models to frequency-domain perturbations, while the partial recovery achieved for SSAA suggests that incorporating speckle-like perturbations during training may further improve stability. Ensemble approaches using architecturally diverse models, rather than multiple augmented copies of a single network, may further reduce correlated failure modes and improve robustness under both spatial- and frequency-domain perturbations [26,27]. Evaluation across other segmentation architectures would further clarify whether the observed defense asymmetry is specific to U-Net or generalizes across models. Beyond mitigation, the observed consistency patterns across stochastic predictions point to a practical role for confidence-based triage mechanisms that flag unstable or low-consensus segmentations for human review [7,9,28]. Such approaches may be particularly important in post-market deployment, where ongoing monitoring of model behaviour and failure patterns is necessary to detect emerging vulnerabilities and support safe clinical use over time.

This study was not without its limitations. The evaluation was conducted on a single dataset with a single model architecture, and generalizability to other datasets, anatomical sites, or architectures cannot be assumed. In addition, the attacks used a fixed query budget of 500, and stronger attacks with larger budgets could potentially overcome the defenses that showed effectiveness. The dataset was acquired at a single institution, and its speckle characteristics may not reflect variability across ultrasound vendors, probe frequencies, and acquisition settings. The use of a standard U-Net without pre-training may also have influenced susceptibility to attack. Although SimBA produced substantially less degradation under the same query budget, indicating that attack formulation contributed to the observed effects within this model, the extent to which adversarial susceptibility depends on the underlying architecture cannot be determined from a single-model evaluation. Attacks were evaluated on the middle frames of the test cine-clips; temporal consistency across consecutive frames was not assessed. The baseline DSC of 0.76 aligns with prior ultrasound segmentation studies [2,29], although the clinical significance of the observed DSC reductions remains unclear. Visual similarity was also assessed quantitatively using SSIM and perturbation norms rather than through a blinded sonographer or radiologist reader study; therefore, human perceptual imperceptibility was not established. Lastly, the defenses were evaluated under a non-adaptive threat model, and adaptive attacks specifically designed to circumvent the tested defenses could be more effective.

## Conclusion

This study demonstrates that the evaluated U-Net deep learning model for thyroid nodule ultrasound segmentation is vulnerable to black-box adversarial perturbations. Spatial-domain attacks were partially mitigated by simple inference-time preprocessing, recovering up to 36% of lost DSC with negligible cost to unperturbed performance, whereas statistically significant DSC recovery was not demonstrated for the frequency-domain attack. These findings suggest an attack-dependent difference in defense effectiveness under the evaluated conditions. Future work should focus on training-level and architectural approaches to improve robustness, and on evaluating these strategies across additional ultrasound tasks to support safe and reliable clinical deployment.

## Supporting information

S1 Table Quantitative image-similarity metrics. DSC = Dice similarity coefficient; SD = standard deviation; SSIM = Structural Similarity Index (1.0 = identical images); L_2_ = Euclidean norm of the perturbation; L_∞_ = maximum per-pixel perturbation magnitude.
(DOCX)

S2 Table Simple Black-box Adversarial Attack (SimBA) baseline comparison. Panel A reports attack effectiveness for SimBA alongside SSAA and FDUA under the same query budget of 500. Panel B reports defense effectiveness against SimBA, assessed by one-sided paired Wilcoxon signed-rank test with Bonferroni correction (adjusted α = 0.0167 for three comparisons). No defense achieved statistical significance. DSC = Dice similarity coefficient; SSIM = Structural Similarity Index; SD = standard deviation; CI = bootstrap 95% confidence interval (1,000 resamples).
(DOCX)

S3 Table Recovery rates across metrics. Recovery rate = (mitigated metric − undefended metric) divided by (unperturbed metric − undefended metric). Values are expressed as percentages. Negative precision recovery indicates a shift toward over-segmentation. DSC = Dice similarity coefficient; IoU = Intersection over Union; SSAA = Structured Speckle Amplification Attack; FDUA = Frequency-Domain Ultrasound Attack.
(DOCX)

S1 Fig Frequency spectrum analysis of unperturbed, FDUA-attacked, and denoised images. Top row = spatial domain. Bottom row = log-magnitude Fourier spectrum. FDUA = Frequency-Domain Ultrasound Attack.
(TIF)

S2 Fig Stochastic ensemble consistency maps showing per-pixel agreement across K = 5 augmentation copies. Green = high agreement; Red = low agreement; Dice = Dice similarity coefficient; SSAA = Structured Speckle Amplification Attack; FDUA = Frequency-Domain Ultrasound Attack.
(TIF)

S3 Fig Grouped boxplot of adversarial dice similarity coefficient by attack and defense condition. Diamond markers indicate group means. Dashed grey line represents baseline model performance. DSC = Dice similarity coefficient; SSAA = Structured Speckle Amplification Attack; FDUA = Frequency-Domain Ultrasound Attack.
(TIF)

S4 Fig Dice recovery rate (%) for each defense against SSAA and FDUA. DSC = Dice similarity coefficient; SSAA = Structured Speckle Amplification Attack; FDUA = Frequency-Domain Ultrasound Attack.
(TIF)
