# Supplementary material for: Adversarial robustness of deep learning-based thyroid nodule segmentation in ultrasound
Source: PLOS Digit Health. 2026 Nov 4;5(11):e0001797. doi: 10.1371/journal.pdig.0001797 (PMC13734574; doi:10.1371/journal.pdig.0001797)
Supplement: Supplementary file 1 [file pdig.0001797.s001.docx]

**S1 Table**. Quantitative image-similarity metrics. DSC = Dice similarity coefficient; SD = standard deviation; SSIM = Structural Similarity Index (1.0 = identical images); L_2_ = Euclidean norm of the perturbation; L_∞_ = maximum per-pixel perturbation magnitude.

| **Attack** | **DSC Drop** | **SD** | **SSIM** | **L_2_** | **L_∞_** |
| --- | --- | --- | --- | --- | --- |
| SSAA | 0.29 | 0.20 | 0.94 | 5.9 | 0.27 |
| FDUA | 0.11 | 0.09 | 0.82 | 5.0 | 0.09 |
