# Supplementary material for: Adversarial robustness of deep learning-based thyroid nodule segmentation in ultrasound
Source: PLOS Digit Health. 2026 Nov 4;5(11):e0001797. doi: 10.1371/journal.pdig.0001797 (PMC13734574; doi:10.1371/journal.pdig.0001797)
Supplement: Supplementary file 2 [file pdig.0001797.s002.docx]

**S2 Table.** Simple Black-box Adversarial Attack (SimBA) baseline comparison. Panel A reports attack effectiveness for SimBA alongside SSAA and FDUA under the same query budget of 500. Panel B reports defense effectiveness against SimBA, assessed by one-sided paired Wilcoxon signed-rank test with Bonferroni correction (adjusted α = 0.0167 for three comparisons). No defense achieved statistical significance. DSC = Dice similarity coefficient; SSIM = Structural Similarity Index; SD = standard deviation; CI = bootstrap 95% confidence interval (1,000 resamples).

***Panel A.*** Attack comparison under equal query budget (500)

| **Attack** | **DSC Drop** | **SD** | **SSIM** | **L2** | **L∞** | **Queries** |
| --- | --- | --- | --- | --- | --- | --- |
| SimBA | 0.01 | 0.01 | >0.99 | 0.29 | 0.05 | 500 |
| SSAA | 0.29 | 0.20 | 0.94 | 5.90 | 0.27 | 500 |
| FDUA | 0.11 | 0.09 | 0.82 | 5.00 | 0.09 | 500 |

***Panel B.*** Defense effectiveness against SimBA

| **Defense** | **Δ DSC** | **95% CI** | **p-value** | **Cohen's d** |
| --- | --- | --- | --- | --- |
| Randomized Preprocessing | +0.01 | [−0.02, +0.03] | 0.42 | +0.08 |
| Deterministic Denoising | +0.00 | [−0.02, +0.03] | 0.63 | +0.04 |
| Stochastic Ensemble | +0.02 | [−0.01, +0.04] | 0.34 | +0.23 |
