# Supplementary material for: Adversarial robustness of deep learning-based thyroid nodule segmentation in ultrasound
Source: PLOS Digit Health. 2026 Nov 4;5(11):e0001797. doi: 10.1371/journal.pdig.0001797 (PMC13734574; doi:10.1371/journal.pdig.0001797)
Supplement: Supplementary file 3 [file pdig.0001797.s003.docx]

**S3 Table.** Recovery rates across metrics. Recovery rate = (mitigated metric − undefended metric) divided by (unperturbed metric − undefended metric). Values are expressed as percentages. Negative precision recovery indicates a shift toward over-segmentation. DSC = Dice similarity coefficient; IoU = Intersection over Union; SSAA = Structured Speckle Amplification Attack; FDUA = Frequency-Domain Ultrasound Attack.

| **Attack** | **Defense** | **DSC** | **IoU** | **Precision** | **Recall** | **Pixel Accuracy** |
| --- | --- | --- | --- | --- | --- | --- |
| SSAA | Randomized Preprocessing | 29.29% | 27.93% | 8.23% | 35.66% | 15.29% |
| SSAA | Denoising | 35.57% | 33.48% | −4.63% | 35.09% | 15.76% |
| SSAA | Stochastic Ensemble | 28.24% | 26.73% | 8.45% | 26.52% | 18.00% |
| FDUA | Randomized Preprocessing | 16.92% | 18.04% | −23.18% | 62.60% | 5.84% |
| FDUA | Denoising | 20.60% | 21.23% | −29.26% | 87.65% | 2.91% |
| FDUA | Stochastic Ensemble | 8.06% | 9.42% | −19.97% | 23.72% | −5.22% |
